# Food Insecurity and Appetitive Traits: A Prospective Analysis in Generation XXI Cohort

**DOI:** 10.1002/eat.24530

**Published:** 2025-08-21

**Authors:** Alexandra Costa, Marion M. Hetherington, Andreia Oliveira

**Affiliations:** ^1^ EPIUnit ITR Instituto de Saúde Pública da Universidade do Porto Porto Portugal; ^2^ School of Psychology University of Leeds Leeds UK; ^3^ Departamento de Saúde Pública e Ciências Forenses e Educação Médica, Faculdade de Medicina Universidade do Porto Porto Portugal

**Keywords:** adolescence, appetite, cohort studies, eating behavior, food insecurity

## Abstract

**Objective:**

To evaluate the association between food insecurity at age 10 and appetitive traits at age 13.

**Methods:**

The analysis included a subsample of participants from the Generation XXI birth cohort with available data on the variable of interest. Household food security status was assessed using the US Household Food Security Survey Module, completed by the primary caregiver. In addition, children's perceptions of food security at age 10 were evaluated using the Self‐Administered Food Security Survey Module. Appetitive traits were assessed at age 13 by the Children's Eating Behavior Questionnaire. Linear regression models, adjusted for socio‐demographic variables, estimated the associations between food insecurity and appetitive traits (*n* = 2495).

**Results:**

At age 10, 5.8% of participants lived in food‐insecure households. These participants showed greater responsiveness to food ($\hat{\beta }$ = 0.27, 95% CI 0.13, 0.42), enjoyed foods more ($\hat{\beta }$ = 0.22, 95% CI 0.08, 0.35), had a higher desire to drink ($\hat{\beta }$ = 0.26, 95% CI 0.14, 0.37), and more emotional overeating ($\hat{\beta }$ = 0.28, 95% CI 0.15, 0.41) and emotional undereating ($\hat{\beta }$ = 0.15, 95% CI 0.01, 0.28) 3 years later compared to those from food‐Secure households. Analysis using child‐reported perceived food security status showed similar relationships, except for emotional undereating, where there was no significant association.

**Discussion:**

Experience of food insecurity in childhood was associated with increased food approach appetitive traits at age 13, reflecting amplification of an avid appetite phenotype and acquisition of emotional eating. These results highlight food insecurity's potential long‐term health consequences during critical developmental periods.

**Public Significance Statement:**

Children who experienced food insecurity at age 10 showed a stronger response to food, a greater interest in eating, and a tendency to eat in response to negative emotions by age 13. These behaviors are known to increase the risk of obesity. Early food insecurity can have long‐lasting effects on children's health and eating behaviors.

**Summary**

- We evaluated the associations between food insecurity at age 10 and appetitive traits 3 years later using prospective data from a birth cohort.
- Food insecurity in childhood was associated with increased responsiveness to food cues, general interest in food, desire to drink, and tendency to eat in response to negative emotions.
- These appetitive traits are linked to an elevated risk of obesity, highlighting the potential lasting effects of experiencing food insecurity during key developmental stages.

## 1 Introduction

Food insecurity, defined as the limited or uncertain availability of nutritionally adequate food or the inability to acquire food in a socially acceptable way, remains a serious public health concern, even in high‐income countries (Seligman and Schillinger 2010). It adversely affects multiple health domains and is especially detrimental during childhood, a critical period when adequate nutrition is essential for growth and development (de Oliveira et al. 2020). Although the relationship is complex, food insecurity has been associated with an increase in obesity risk in both children and adults, a phenomenon often referred to as the “food insecurity‐obesity paradox” (Nettle et al. 2017; Seligman and Schillinger 2010).

Food insecurity impacts dietary intake in numerous ways, often leading to reliance on inexpensive, palatable, energy‐dense foods to meet energy requirements (Hanson and Connor 2014). Beyond immediate dietary impacts, it may also cause lasting alterations in eating behaviors through different mechanisms (Kosmas et al. 2023; Tester et al. 2020). For instance, individuals experiencing food insecurity often fluctuate between periods of food availability and scarcity, known as the “feast‐or‐famine” cycle, which can contribute to dysregulated eating behaviors (Dinour et al. 2007). Additionally, the stress and anxiety induced by uncertainty around food can further negatively influence eating behaviors (Kosmas et al. 2023). Within the spectrum of disordered eating, research has consistently shown an association between food insecurity and binge eating and compensatory behaviors (Bidopia et al. 2023). Studies have also suggested that food insecurity may foster the development of obesogenic behaviors in childhood, including eating in the absence of hunger and food approach appetitive traits, characterized by an avid appetite and a strong interest in food (Eagleton et al. 2022; Harshman et al. 2021; McCurdy et al. 2022; Pickard et al. 2023). It has been demonstrated that some appetitive traits are highly heritable (e.g., Satiety Responsiveness, Fussy Eating, and Food Responsiveness) (Llewellyn et al. 2023). However, emotional eating appears to be acquired in early life as a function of experience (Herle et al. 2018). Therefore, the experience of food insecurity could amplify existing behavioral susceptibility to an avid appetite and lead to the tendency to overeat and undereat in response to stressful situations.

Some inconsistencies across studies complicate our understanding of how food insecurity may affect later eating behavior (Eagleton et al. 2022; McCurdy et al. 2022; Oberle et al. 2019; Pickard et al. 2023). For example, while some studies link food insecurity with appetitive traits, others report no such associations (Eagleton et al. 2022; McCurdy et al. 2022; Oberle et al. 2019; Pickard et al. 2023). In addition, most research has concentrated on young children, with the effects on older age groups unknown. There are also no studies considering children's perceptions of food insecurity, which may differ from those of their caregivers. For instance, caregivers may shield children from food shortages or, in other cases, underreport food insecurity due to stigma, while children may still perceive and be affected by it (Barry et al. 2022). Further research is necessary to address these gaps, particularly studies with a prospective design and a well‐characterized population‐based sample. These findings could enhance our understanding of the broader consequences of food insecurity and whether it influences appetitive traits in ways that can potentially promote overeating and obesity. Accordingly, the aim of this study was to investigate the association between food insecurity at age 10 and appetitive traits at age 13. We hypothesized that children in food‐insecure households would exhibit increased food approach tendencies linked to an avid appetite.

## 2 Methods

### 2.1 Study Population

**FIGURE 1:**
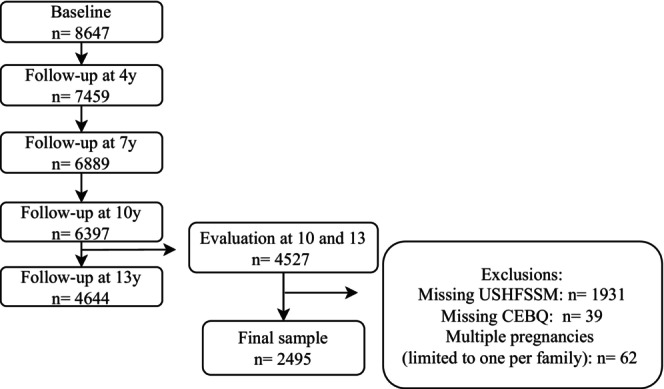
Flowchart of participants' selection from Generation XXI birth cohort. CEBQ: Children's Eating Behavior Questionnaire, USHFSSM: US Household Food Security Survey Module.

This study used data from Generation XXI, a prospective population‐based birth cohort study assembled in Porto, Portugal (Alves et al. 2012; Larsen et al. 2013). A total of 8647 liveborn infants were enrolled between April 2005 and August 2006 in public maternity wards from Porto. All families were invited to attend evaluations when children were aged 4, 7, 10, and 13 years old (participation proportion of 86%, 80%, 76%, and 54%–early stopped due to the COVID‐19 pandemic, respectively). As part of the 10‐year‐of‐age follow‐up evaluation, a subsample was invited to a food security status assessment (*n* = 2942). For this study, we considered participants who had data simultaneously on food security status at age 10 and appetitive traits at age 13 (*n* = 2557). In cases of multiple births, we randomly selected one child per family, resulting in a study sample of 2495 participants (Figure 1). Baseline comparisons of specific maternal characteristics between this study sample and the remaining cohort (*n* = 6090) revealed that mothers in our sample were, on average, older (*M* = 31.0, SD = 4.9 vs. *M* = 28.5, SD = 6.2 years; *p* < 0.001) and had more years of education (*M* = 11.6, SD = 4.2 vs. *M* = 10.0, SD = 4.2 years; *p* < 0.001). However, Cohen's d‐effect size values were 0.44 and 0.38, respectively, suggesting these significant differences may be due to the large sample size rather than systematic differences between these two groups (Husted et al. 2000).

Generation XXI cohort was approved by the University of Porto Medical School/S. João Hospital Centre Ethics Committee, except for the 13‐year follow‐up, which was approved by the ISPUP Ethics Committee. All study phases complied with the Ethical Principles for Medical Research Involving Human Subjects expressed in the Declaration of Helsinki. All procedures were explained to participants, and informed consent was signed by one of the parents or legal guardians (at 13 years, it was also signed by the participants). Baseline evaluation was additionally approved by the Data Protection National Commission, and the study follows the present EU General Data Protection Regulation under close supervision of the Data Protection Office of ISPUP.

### 2.2 Data Collection

Data were collected through in‐person, face‐to‐face interviews conducted by trained researchers or through self‐completed questionnaires. Children's anthropometrics were measured at each follow‐up, following standardized procedures, and age‐ and sex‐specific BMI z‐scores were calculated and categorized according to the WHO criteria (< −2 SD for underweight, −2 to +1 SD for normal weight, > +1 SD for overweight, and > +2 SD for obesity) (de Onis et al. 2012).

### 2.3 Food Security Status

Food security status was assessed using the US Household Food Security Survey Module (USHFSSM) (2012 version), validated for the Portuguese population (Maia et al. 2022; United States Department of Agriculture 2012). Parents or primary caregivers completed this module in a face‐to‐face interview during the child's 10‐year follow‐up evaluation. The USHFSSM includes 18 questions addressing the food situation during the previous 12 months, such as whether the household could afford the necessary food or ate less due to financial constraints. Response scores were categorized as follows: a score of 0 indicated high food security, 1–2 marginal food security, 3–7 low food security, and 8–18 very low food security, in alignment with the USDA's classification guidelines for households with at least one child (United States Department of Agriculture 2012). For analysis, households with high or marginal food security were classified as food secure, and those with low or very low were classified as food insecure (United States Department of Agriculture 2012). Additionally, as a complementary analysis, we treated food insecurity as a discrete variable ranging from 1 to 18 (sum score of affirmative responses).

Children's food insecurity experiences may differ from adults in the same household. Therefore, the Self‐Administered Food Security Survey Module (SAFSSMC), validated for use in the Portuguese population (Coleman‐Jensen and Nord 2006; Maia et al. 2020), was self‐administered separately to children. The SAFSSMC includes 9 questions about the food situation in the household in the previous month (e.g., *Did you worry that food at home would run out before your family got money to buy more*?), each with three response options (“a lot”, “sometimes”, and “never”). Children were classified as food secure or insecure based on the classification framework established in the validated Portuguese version of the SAFSSMC (Maia et al. 2020).

### 2.4 Appetitive Traits

Appetitive traits were assessed with the Children's Eating Behavior Questionnaire (CEBQ) (Wardle et al. 2001), a parent‐reported questionnaire with 35 items that evaluates eight subscales, frequently grouped into food approach traits: Food Responsiveness (5 items—e.g., *My child's always asking for food*.), Enjoyment of Food (4 items—e.g., *My child loves food*), Emotional Overeating (4 items—e.g., *My child eats more when anxious*.), Desire to Drink (3 items—e.g., *If given the chance, my child would always be having a drink*.) and food avoidance: Satiety Responsiveness (5 items—e.g., *My child gets full up easily*.), Slowness in Eating (4 items—e.g., *My child eats slowly*.), Emotional Undereating (4 items—e.g., *My child eats less when s/he is upset*.), and Food Fussiness (6 items—e.g., *My child decides that s/he doesn't like food, even without tasting it*.). All items are scored on a 5‐point Likert scale as never, rarely, sometimes, often, or always. For questionnaires with < 50% of missing items (around 3% of the sample), data were imputed by a replacement for the average of the remaining questions within each subscale of the participant. The CEBQ was validated in children from our cohort, showing good psychometric properties (Albuquerque et al. 2017). In this sample, at 13 years old, McDonald's omega coefficients varied from 0.72 to 0.83.

### 2.5 Covariates

We selected a comprehensive set of covariates based on previous studies (Barry et al. 2022; Maia and Santos 2022), including child's sex, maternal age and years of education, household monthly income, family structure, and neighborhood socioeconomic deprivation at 10 years of age. Household monthly income was collected as a categorical variable and classified into two groups: low income (≤ 1000 €) and intermediate to high income (≥ 1001 €); the intermediate (≥ 1001€–2000 €) and high‐income (> 2000 €) categories were combined due to the low prevalence of food insecurity among high‐income households. Family structure was determined by asking with whom the child lived. Neighborhood socioeconomic deprivation was assessed using the European Deprivation Index, a multivariable index developed to classify small areas according to their level of socioeconomic deprivation (the first tercile corresponds to least deprived areas) (Ribeiro et al. 2018).

### 2.6 Statistical Analysis

We used linear regression models to assess the association between food security status at age 10 (food secure [as reference] vs. food insecure) and appetitive traits at age 13. As a complementary analysis, food insecurity was also treated as an interval‐scaled variable. Estimates were expressed using regression coefficients ($\hat{\beta }$) and 95% confidence intervals (95% CI). Standardized regression coefficients ($\hat{\beta }$) and *R* ^2^ values were reported. To minimize bias from missing data and attrition, we assumed missing at random and applied multiple imputation using the Multivariate Imputation by Chained Equations (MICE) package. Missing covariates were imputed for < 3% of cases for monthly disposable income and were nearly negligible for other variables. We tested interactions between household income categories and food insecurity by including an interaction term (income × food security status) in the models. However, as the interaction was non‐significant, it was removed. Nonetheless, we ran models stratified by income categories as a sensitivity analysis. Additionally, we tested the association between food security status reported by the children (instead of the primary caregiver) and appetitive traits.

We checked linear regression assumptions using appropriate visualizations. Multicollinearity between predictors was assessed through the tolerance and variance inflation factor (VIF), with no collinearity detected (VIF > 5). We used Cohen's Kappa (*κ*) to test the agreement between caregivers' and childre's reports of food insecurity. All statistical analyses were performed in R 4.3.0, with statistical significance set at *p* < 0.05 (two‐sided).

## 3 Results

The characteristics of the study sample are presented in Table 1. The prevalence of household food insecurity at age 10 was 5.8% (*n* = 145). Among participants from food‐insecure households, 63.8% were defined as having a low income, 43.4% resided in the most deprived neighborhoods, and 50.3% had overweight or obesity.

**TABLE 1:** Characteristics of study participants from the Generation XXI birth cohort, for the full sample and stratified by food security status when children were aged 10.

	Total	Food secure^a^	Food insecure^a^	*p*
	(*n* = 2495)	(*n* = 2350)	(*n* = 145)	
Family characteristics				
Maternal age at baseline (y), mean (SD)	30.4 (4.9)	30.4 (4.9)	30.3 (5.5)	0.850
Maternal education at baseline (y), mean (SD)	11.6 (4.2)	11.8 (4.2)	8.1 (3.5)	< 0.001
Household monthly income (euros) at age 10, *n* (%)				
Low (≤ 1000)	569 (23.4)	479 (20.9)	90 (63.8)	< 0.001
Intermediate to high ≥ (1001)	1866 (76.6)	1815 (79.1)	51 (36.2)	
Family structure at age 10, *n* (%)				
Both parents	2042 (83.2)	1948 (84.2)	94 (66.7)	< 0.001
Only mother or father/neither mother nor father	413 (16.8)	366 (15.8)	47 (32.3)	
Neighborhood deprivation at age 10, *n* (%)				
First tertile (least deprived)	947 (38.0)	916 (39.0)	31 (21.4)	< 0.001
Second tertile	865 (34.7)	814 (34.6)	51 (35.2)	
Third tertile (most deprived)	683 (27.3)	620 (26.4)	63 (43.4)	
Child characteristics				
Sex, *n* (%)				
Female	1165 (46.7)	1096 (46.6)	69 (47.6)	0.824
Male	1330 (53.3)	1254 (53.4)	76 (52.4)	
Child's weight indicators				
BMI z‐score at 10, mean (SD)^b^	0.67 (1.2)	0.65 (1.2)	1.0 (1.2)	< 0.001
BMI status at age 10, *n* (%)^b^				
Underweight	27 (1.1)	27 (1.2)	0 (0.0)	< 0.001
Normal weight	1432 (57.8)	1368 (58.5)	64 (45.7)	
Overweight	661 (26.7)	614 (26.3)	47 (33.6)	
Obesity	357 (14.4)	328 (14.0)	29 (20.7)	
BMI z‐score at 13, mean (SD)^b^	0.42 (1.2)	0.40 (1.2)	0.82 (1.2)	< 0.001
BMI status at age 13, *n* (%)^b^				
Underweight	56 (2.4)	56 (2.4)	0 (0.0)	< 0.001
Normal weight	1625 (66.1)	1553 (66.1)	72 (49.7)	
Overweight	583 (22.7)	534 (22.7)	49 (16.5)	
Obesity	231 (8.8)	207 (8.8)	24 (33.8)	

Abbreviations: BMI z‐scores, body mass index z‐score; SD, standard deviation; y, years.

^a^ Assessed by the US Household Food Security Survey Module (United States Department of Agriculture 2012), completed by a parent or primary caregiver.

^b^ Classification according to the WHO criteria (de Onis et al. 2012). *N* varies between 2495 and 2435 due to missing values.

**FIGURE 2:**
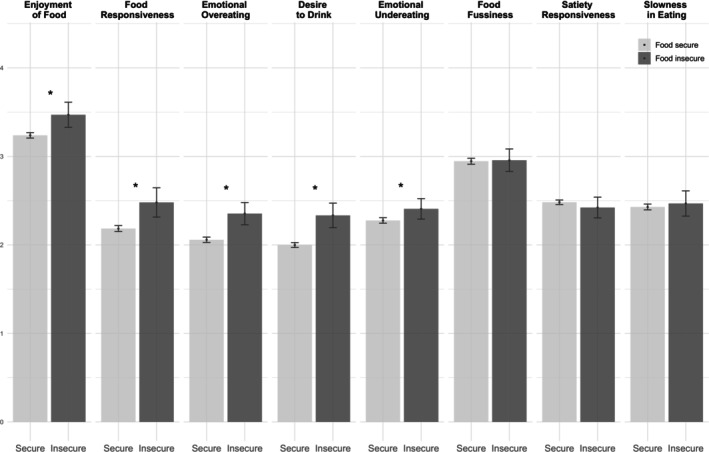
Mean scores of appetitive traits at age 13 by food security status reported by caregivers at age 10. Bars represent 95% confidence intervals. *Significant results (*p* < 0.05), group differences evaluated with *t*‐test.

At age 13, mean scores for several appetitive traits differed significantly between participants from food‐secure and food‐insecure households (Figure 2). Multivariable linear regression models (Table 2) showed that participants from food‐insecure households had higher scores on Food Responsiveness, Enjoyment of Food, Desire to Drink, Emotional Overeating, and Emotional Undereating than those from food‐secure households. No associations were found for Satiety Responsiveness, Slowness in Eating, or Food Fussiness. Considering food insecurity as a discrete variable (Table S1), higher scores were associated with greater Food Responsiveness ($\hat{\beta }$ = 0.06, 95% CI 0.03, 0.08), Enjoyment of Food ($\hat{\beta }$ = 0.04, 95% CI 0.02, 0.07), Desire to drink ($\hat{\beta }$ = 0.06, 95% CI 0.04, 0.08), and Emotional Overeating ($\hat{\beta }$ = 0.06, 95% CI 0.04, 0.08).

**TABLE 2:** Associations between food security status reported by parents when children were aged 10 and appetitive traits at age 13.

Appetitive traits at age 13		Food insecure at age 10^a^				*R* ^2^
		$\hat{\beta }$ (95% CI)	*p*	Intercept (95% CI)	Standardized $\hat{\beta }$ (95% CI)	
Food Responsiveness	Crude	**0.30 (0.16, 0.45)**	**< 0.001**	**2.18 (2.15, 2.22)**	**0.09 (0.05, 0.12)**	0.01
	Adjusted^b^	**0.27 (0.13, 0.42)**	**< 0.001**	**2.83 (2.61, 3.05)**	**0.08 (0.04, 0.12)**	0.03
Enjoyment of Food	Crude	**0.24 (0.11, 0.37)**	**< 0.001**	**3.24 (3.21, 3.27)**	**0.07 (0.03, 0.11)**	0.01
	Adjusted^b^	**0.22 (0.08, 0.35)**	**0.002**	**3.64 (3.43, 3.84)**	**0.07 (0.02, 0.11)**	0.01
Desire to Drink	Crude	**0.35 (0.23, 0.47)**	**< 0.001**	**2.00 (1.97, 2.03)**	**0.12 (0.08, 0.16)**	0.01
	Adjusted^b^	**0.26 (0.14, 0.37)**	**< 0.001**	**2.64 (2.46, 2.83)**	**0.09 (0.05, 0.13)**	0.04
Emotional Overeating	Crude	**0.30 (0.18, 0.42)**	**< 0.001**	**2.06 (2.03, 2.09)**	**0.12 (0.06, 0.13)**	0.09
	Adjusted^b^	**0.28 (0.15, 0.41)**	**< 0.001**	**2.53 (2.34, 2.73)**	**0.09 (0.05, 0.13)**	0.03
Emotional Undereating	Crude	**0.13 (0.01, 0.26)**	**0.048**	**2.28 (2.25, 2.31)**	**0.04 (0.01, 0.08)**	0.01
	Adjusted^b^	**0.15 (0.01, 0.28)**	**0.003**	**2.34 (2.13, 2.54)**	**0.04 (0.01, 0.09)**	0.02
Satiety Responsiveness	Crude	−0.08 (−0.18, 0.03)	0.148	**2.48 (2.46, 2.51)**	−0.02 (−0.07, 0.01)	0.01
	Adjusted^b^	−0.04 (−0.15, 0.07)	0.417	**2.43 (2.26, 2.60)**	−0.02 (−0.06, 0.02)	0.02
Slowness in Eating	Crude	0.06 (−0.08, 0.20)	0.397	**2.43 (2.40, 2.46)**	0.02 (−0.02, 0.06)	0.00
	Adjusted^b^	0.06 (−0.08, 0.20)	0.431	**2.64 (2.42, 2.86)**	0.02 (−0.02, 0.06)	0.02
Food Fussiness	Crude	0.02 (−0.12, 0.16)	0.776	**2.95 (2.91, 2.98)**	0.01 (−0.03, 0.05)	0.00
	Adjusted^b^	−0.01 (−0.16, 0.13)	0.895	**2.98 (2.75, 3.20)**	0.00 (−0.04, 0.04)	0.00

Abbreviations: CI, confidence interval; *R* ^2^, variance explained by the model.

^a^ Food security status assessed by the US Household Food Security Survey Module (United States Department of Agriculture 2012), completed by a parent or primary caregiver; Food secure as the reference category.

^b^ Model adjusted for maternal age, education, monthly disposable income, family structure, neighborhood deprivation, and child's sex. Significant results in bold (*P* < 0.05).

In a sensitivity analysis stratified by household income (Table S2), no association was observed for Emotional Undereating at both strata; the association with Enjoyment of Food was non‐significant in the intermediate/high‐income group. The other associations remained consistent with those observed in the full sample.

An analysis using child‐reported food insecurity status (Table S3) showed a higher prevalence of food insecurity (9.0%, *n* = 225) than parental reported food insecurity. Agreement between children's and caregivers' reports was low (Cohen's Kappa (*κ*) = 0.12), with high specificity (95%) but low sensitivity (15%). Despite these differences, associations with appetite traits were comparable, with food insecurity being consistently associated with higher scores on all food approach traits; only the association with Emotional Undereating lost statistical significance (see Table S4).

## 4 Discussion

This study found an association between food insecurity at age 10 and high scores on food approach appetitive traits at age 13, suggesting that food insecurity may amplify existing behavioral susceptibility to an avid appetite and play a role in the development of emotional eating. Perception of food insecurity between caregivers and children differed. However, the perception of food security status by the caregivers or by the children confirmed the relationship between food insecurity and avid appetite tendencies, except for Emotional Undereating.

As hypothesized, participants from food‐insecure households showed significantly higher scores of Food Responsiveness, Enjoyment of Food, Desire to Drink, and Emotional Overeating than those from food‐secure households—traits associated with increased obesity risk (Kininmonth et al. 2021). Additionally, results showed that as the degree of food insecurity increased, scores on these traits also increased. Our results aligned with prior research on younger children from low‐income backgrounds; in children aged 3–5, food insecurity was associated with increased Food Responsiveness and Emotional Overeating but not with food avoidance traits (Eagleton et al. 2022). Similarly, another study reported elevated Food Responsiveness and Enjoyment of Food scores in preschoolers from low‐income families experiencing food insecurity (McCurdy et al. 2022). A study analyzing patterns of appetitive traits in young children also found a higher proportion of children experiencing food insecurity in the “avid eater” profile compared to the others (Pickard et al. 2023). In contrast, in a study on obesity treatment‐seeking children, food insecurity was only significantly associated with Desire to Drink among all CEBQ traits (Oberle et al. 2019). This divergence may be due to this sample comprising mostly children living with severe obesity. These children may exhibit more homogenous appetitive traits—typically increased food approach and reduced food avoidance—leading to limited variability that may reduce the likelihood of detecting associations.

Several mechanisms may explain how food insecurity acts on appetitive traits, particularly those that are learned. One explanation is that food serves as a coping mechanism to manage psychosocial stress induced by food shortage (Martin et al. 2016; Miller and Lumeng 2018). Qualitative studies indicate that children in food‐insecure households often report feelings of worry, sadness, and anger about their household's food situation (Harvey 2016; Leung et al. 2020); some individuals may use eating as a strategy to deal with these negative emotions (i.e., Emotional Overeating or Undereating) (Kosmas et al. 2023; Miller and Lumeng 2018). Additionally, chronic stress due to food insecurity may elevate cortisol levels, potentially disrupting long‐term insulin and leptin regulation, which increases appetite and the desire for highly palatable foods (Adam and Epel 2007; Miller and Lumeng 2018). Another mechanism involves the so‐called “feast‐or‐famine” cycle. In food‐insecure households, periodic scarcity may lead to cycles of overeating when food is available, potentially interfering with appetite regulation (Bidopia et al. 2023; Dinour et al. 2007). This pattern is evidenced in traits such as Food Responsiveness (frequent requests for food, eating too much if allowed, and eating beyond hunger) and Enjoyment of Food (anticipation of meals and elevated interest in food).

Among food avoidance traits, only Emotional Undereating showed a significant association with food insecurity. However, this association was relatively weak and only significant when considering food security status reported by the parents. Nevertheless, previous studies have shown that some individuals may have an inherent tendency to either under‐ or overeat in response to negative emotions, with specific stressors influencing the direction of this response (Albuquerque et al. 2017). The lack of association with the other food avoidance traits (Satiety Responsiveness, Slowness in Eating, and Food Fussiness) suggests that, for example, factors such as genetic predisposition or early life determinants may play a more substantial role in shaping these traits than the experience of food insecurity (Albuquerque et al. 2017; Warkentin et al. 2022).

It is crucial to consider other exposures related to the environment in which children live. For instance, factors such as maternal education and socioeconomic status have been linked to increased food approach traits (Costa et al. 2024). The broader food environment may also play a role, as children in more deprived areas often have greater access to energy‐dense foods (Williamson et al. 2017). In our sample, 43% of food‐insecure households lived in the most deprived areas, compared to just 26% of food‐secure households. Additionally, economic hardship can contribute to parental stress and poor mental health, which may negatively impact parenting and eating environments (Martin et al. 2016). This overall context may interact with children's predisposition and exacerbate the development of obesogenic traits.

We did not find strong evidence of an interaction between income and food insecurity, as most associations were consistent across the full sample and the two income strata, highlighting that the impact of food insecurity was not limited to low‐income status. Indeed, income alone was a relatively crude measure of food security, influenced by many socio‐demographic and contextual factors, such as cost of living, employment stability, household composition, and food access (Maia and Santos 2022; Rabbitt et al. 2024).

Despite the low agreement between child‐ and parent‐reported measures, associations with appetitive traits were consistent across both. This may be explained because, although discrepancies in food insecurity classifications exist, children's and parents' reports are expected to capture the same underlying construct. Additionally, households experiencing severe food insecurity are more likely to be identified by children and parents, and these more extreme cases may drive the observed associations. These results highlight the importance of considering both parents' and children's reports when analyzing food insecurity, as they may offer distinct yet complementary perspectives on the experience of food insecurity.

This study has several key strengths, such as using data from a well‐established, population‐based birth cohort and a prospective design. This is the first study to examine the relationship between childhood food insecurity and appetitive traits in early adolescence. Food insecurity was assessed with the USHFSSM, a widely used tool considered a gold standard, and with the SAFSSMC to capture food insecurity specifically at the child level. The associations with appetitive traits were comparable across both independent measures, which provides additional confidence in the robustness of our findings and suggests that these associations are unlikely to be due to random chance (Type I error). We also conducted stratified analyses by income level, and most associations held consistently across income categories despite a lower prevalence of food insecurity in the intermediate to high‐income group.

Regarding the study's limitations, the parent‐reported nature of the CEBQ may introduce some measurement errors. However, the CEBQ has been previously validated against behavioral measures of eating and has demonstrated strong internal consistency within our population (Albuquerque et al. 2017; Carnell and Wardle 2007). Additionally, the prevalence of household food insecurity in our sample (5.8%) was lower than national estimates of 11.4% for households with children under 18 (Lopes et al. 2018). While differences in study design account for some of this discrepancy, the sample characteristics must be considered. Mothers of participants in our sample tended to be slightly older and more educated than non‐participants, suggesting that our sample may be skewed toward higher socioeconomic status. This selection bias could limit the generalizability of our findings and may have reduced our ability to detect additional associations due to reduced heterogeneity and a lower prevalence of food insecurity. However, this bias is unlikely to compromise the validity of the associations we observed. Lastly, we considered only a single measure of food insecurity, not considering how variations in the severity or duration of food insecurity may differently impact appetitive traits or isolate the impact of changes in appetitive traits in response to changes in food insecurity.

## 5 Conclusion

This study supports the importance of mitigating food insecurity. Our findings suggest an association between food insecurity in childhood and specific appetitive traits in early adolescence—namely, increased responsiveness to food cues, general interest in food, desire to drink, and eat in response to negative emotions. These traits, including those that characterize an avid appetite, are known to be linked to an elevated risk of overeating and, therefore, obesity, highlighting the potential long‐term health consequences of food insecurity during a critical developmental period.

## Author Contributions

**Alexandra Costa:** conceptualization, formal analysis, writing – original draft. **Marion M. Hetherington:** writing – review and editing. **Andreia Oliveira:** conceptualization, methodology, writing – review and editing.

## Conflicts of Interest

The authors declare no conflicts of interest.

## Supporting information


**Table S1:** Associations between food insecurity (discrete variable), reported by the parents at age 10, and appetitive traits at age 13.
**Table S2:** Associations between food security status at age 10, reported by caregivers (food security as reference), and appetitive traits at age 13, stratified by monthly household income.
**Table S3:** Agreement between children and parents reports of food security status at age 10.
**Table S4:** Associations between food security status, reported by the children at age 10, and appetitive traits at age 13.

## Data Availability

The data from Generation XXI are not publicly available due to privacy or ethical restrictions. The data can be made available for research proposals on request to the Generation XXI Executive Committee (generationxxi@ispup.up.pt). Further information about Generation XXI can be obtained via the Generation XXI website [www.geracao21.com] or by emailing generationxxi@ispup.up.pt. Codebook and analytic code will be made available upon request.
