# Supplementary material for: Food Insecurity and Appetitive Traits: A Prospective Analysis in Generation XXI Cohort
Source: Int J Eat Disord. 2025 Aug 21;58(11):2194–202. doi: 10.1002/eat.24530 (PMC12605779; doi:10.1002/eat.24530)
Supplement: Supplementary file 1 — Table S1: Associations between food insecurity (discrete variable), reported by the parents at age 10, and appetitive traits at age 13. Table S2: Associations between food security status at age 10, reported by caregivers (food security as reference), and appetitive traits at age 13, stratified by monthly household income. Table S3: Agreement between children and parents reports of food security status at age 10. Table S4: Associations between food security status, reported by the children at age 10, and appetitive traits at age 13. [file EAT-58-2194-s001.docx]

Supplementary Table 1. Associations between food insecurity (discrete variable), reported by the parents at age 10, and appetitive traits at age 13.

|  |  |  | **Food insecurity at age 10 ^a^** | | |  |
| --- | --- | --- | --- | --- | --- | --- |
| **Appetitive traits at age 13** |  | $\hat{\beta}$ (95% CI) | p-value | Intercept (95% CI) | Standardized $\hat{\beta}$(95% CI) | R^2^ |
| Food Responsiveness | Crude | **0.06 (0.04,0.09)** | **<0.001** | **2.17 (2.14,2.21)** | **0.10 (0.07, 0.14)** | 0.01 |
|  | Adjusted ^b^ | **0.06 (0.03,0.08)** | **<0.001** | **2.73 (2.49,2.97)** | **0.08 (0.04, 0.12)** | 0.03 |
| Enjoyment of Food | Crude | **0.05 (0.02,0.07)** | **<0.001** | **3.23 (3.20,3.26)** | **0.08 (0.04, 0.11)** | 0.01 |
|  | Adjusted ^b^ | **0.04 (0.02,0.07)** | **<0.001** | **3.62 (3.39,3.85)** | **0.07 (0.02, 0.11)** | 0.01 |
| Desire to Drink | Crude | **0.08 (0.06,0.10)** | **<0.001** | **1.98 (1.95,2.01)** | **0.10 (0.06, 0.14)** | 0.02 |
|  | Adjusted ^b^ | **0.06 (0.04,0.08)** | **<0.001** | **2.53 (2.33,2.73)** | **0.09 (0.05, 0.13)** | 0.05 |
| Emotional Overeating | Crude | **0.06 (0.04,0.09)** | **<0.001** | **2.05 (2.02,2.08)** | **0.07 (0.03, 0.11)** | 0.01 |
|  | Adjusted ^b^ | **0.06 (0.04,0.08)** | **<0.001** | **2.43 (2.22,2.65)** | **0.09 (0.05, 0.13)** | 0.03 |
| Emotional Undereating | Crude | **0.03 (0.01,0.06)** | **0.004** | **2.27 (2.24,2.30)** | 0.00 (-0.03, 0.05) | 0.00 |
|  | Adjusted ^b^ | **0.04 (0.01,0.06)** | **0.002** | **2.28 (2.06,2.51)** | 0.04 (0.00, 0.09) | 0.02 |
| Satiety Responsiveness | Crude | -0.02 (-0.03,0.00) | 0.111 | **2.49 (2.46,2.51)** | **-0.05 (-0.09, -0.01)** | 0.00 |
|  | Adjusted ^b^ | -0.01 (-0.03,0.01) | 0.411 | **2.46 (2.27,2.64)** | -0.02 (-0.06, 0.02) | 0.01 |
| Slowness in Eating | Crude | 0.01 (-0.02,0.03) | 0.536 | **2.43 (2.39,2.46)** | -0.02 (-0.06, 0.02) | 0.00 |
|  | Adjusted ^b^ | 0.01 (-0.02,0.03) | 0.536 | **2.67 (2.43,2.91** | 0.02 (-0.02, 0.06) | 0.02 |
| Food Fussiness | Crude | -0.00 (-0.03,0.02) | 0.728 | **2.95 (2.92,2.99)** | -0.02 (-0.06, 0.02) | 0.00 |
|  | Adjusted ^b^ | -0.01 (-0.04,0.01) | 0.329 | **2.94 (2.70,3.19)** | 0.00 (-0.04, 0.04) | 0.00 |

CI: confidence interval. R^2^ variance explained by the model. ^a^ Food security status assessed by the US Household Food Security Survey Module (United States Department of Agriculture, 2012), completed by a parent or primary caregiver (range 1-18). ^b^ Model adjusted for maternal age, education, monthly disposable income, family structure, neighbourhood deprivation, and child’s sex. Significant results in bold (*P*<0.05). N varies between 2483 to 2457.

Supplementary Table 2. Associations between food security status at age 10, reported by caregivers (food security as reference), and appetitive traits at age 13, stratified by monthly household income.

|  | | Low income  (≤1000€) | | | | | Intermediate/High income  (≥1001€) | | | | |
| --- | --- | --- | --- | --- | --- | --- | --- | --- | --- | --- | --- |
| **Appetitive traits at age 13** | | $\hat{\beta}$ (95% CI) | p-value | Intercept  (95% CI) | Standardized  $\hat{\beta}$(95% CI) | R^2^ | $\hat{\beta}$ (95% CI) | p-value | Intercept  (95% CI) | Standardized  $\hat{\beta}$(95% CI) | R^2^ |
| **Food Responsiveness** | Crude | **0.28**  **(0.07, 0.48)** | **0.008** | **2.28**  **(2.21, 2.36)** | **0.10**  **(0.02, 0.18)** | 0.00 | **0.27**  **(0.03, 0.52)** | **0.025** | 2.16  (2.12, 2.19) | **0.05**  **(0.01, 0.10)** | 0.00 |
|  | Adjusted^b^ | **0.32**  **(0.11, 0.52)** | **0.003** | **2.87**  **(2.37, 3.38)** | **0.12**  **(0.04, 0.20** | 0.05 | **0.25**  **(0.03, 0.47)** | **0.023** | 2.62  (2.34,2.90) | **0.05**  **(0.01, 0.10)** | 0.01 |
| **Enjoyment of Food** | Crude | **0.23**  **(0.05, 0.41)** | **0.014** | **3.28**  **(3.21, 3.35)** | **0.10**  **(0.01, 0.18)** | 0.00 | **0.23**  **(0.01, 0.45)** | **0.040** | 3.23  (3.19,3.26) | **0.05**  **(0.00, 0.09)** | 0.00 |
|  | Adjusted^b^ | **0.26**  **(0.08, 0.45)** | **0.007** | **3.82**  **(3.36, 4.26)** | 0.11  (0.03, 0.19) | 0.02 | 0.20  (-0.01, 0.41) | 0.053 | 3.55  (3.29,3.81) | 0.04  (0.00, 0.09) | 0.01 |
| **Desire to Drink** | Crude | **0.23**  **(0.06, 0.40)** | **0.006** | **2.11**  **(2.05, 2.18)** | **0.11**  **(0.03, 0.19)** | 0.01 | **0.33**  **(0.13, 0.54)** | **<0.001** | 1.9  (11.94,2.00) | **0.09**  **(0.05, 0.14)** | 0.00 |
|  | Adjusted^b^ | **0.17**  **(0.01, 0.34)** | **0.043** | **2.73**  **(2.31, 3.14)** | **0.08**  **(0.01, 0.17** | 0.05 | **0.31**  **(0.11, 0.51)** | **<0.001** | 2.51  (2.28,2.73) | **0.08**  **(0.04, 0.13)** | 0.03 |
| **Emotional Overeating** | Crude | **0.25**  **(0.07, 0.43)** | **0.008** | **2.13**  **(2.06, 2.21)** | **0.11**  **(0.03, 0.19)** | 0.01 | **0.31**  **(0.10, 0.54)** | **0.003** | 2.04  (2.01,2.07) | **0.07**  **(0.02, 0.11)** | 0.00 |
|  | Adjusted^b^ | **0.25**  **(0.07, 0.44)** | **0.008** | **2.49**  **(2.03, 2.94)** | **0.11**  **(0.03, 0.19)** | 0.03 | **0.32**  **(0.11, 0.52)** | **0.003** | 2.36  (2.11,2.60) | **0.07**  **(0.02, 0.11)** | 0.02 |
| **Emotional Undereating** | Crude | 0.09  (0.09, 0.27) | 0.341 | **2.30**  **(2.23, 2.37)** | 0.04  (-0.04, 0.13) | 0.00 | 0.08  (-0.14, 0.31) | 0.132 | 2.27  (2.24,2.31) | 0.03  (-0.01, 0.08) | 0.00 |
|  | Adjusted^b^ | 0.08  (-0.10, 0.26) | 0.419 | **2.16**  **(1.72, 2.61)** | 0.04  (-0.05, 0.12) | 0.00 | 0.12  (-0.11, 0.34) | 0.062 | 2.27  (2.01,2.53) | 0.04  (0.00, 0.09 | 0.02 |
| **Satiety Responsiveness** | Crude | -0.13  (-0.27, 0.01) | 0.074 | **2.46**  **(2.40, 2.51)** | -0.07  (-0.15, 0.01) | 0.00 | 0.01  (-0.17, 0.19) | 0.793 | 2.49  (2.46,2.52) | 0.00  (-0.04, 0.05) | 0.00 |
|  | Adjusted^b^ | -0.13  (-0.28, 0.02) | 0.079 | **2.42**  **(2.06, 2.78)** | -0.07  (-0.16, 0.01) | 0.00 | 0.05  (-0.13, 0.24) | 0.581 | 2.45  (2.23,2.66) | 0.00  (-0.03, 0.06) | 0.01 |
| **Slowness in Eating** | Crude | 0.01  (-0.18, 0.19) | 0.975 | **2.43**  **(2.35, 2.50)** | 0.00  (-0.07, 0.09) | 0.00 | 0.06  (-0.17, 0.30) | 0.230 | 2.43  (12.39,2.47) | 0.03  (-0.02, 0.07) | 0.00 |
|  | Adjusted^b^ | 0.01  (-0.19, 0.19) | 0.973 | **2.46**  **(1.99, 2.94)** | 0.00  (-0.08, 0.09) | 0.00 | 2.74  (2.46,3.01) | 0.242 | 0.03  (-0.02,0.07) | 0.00  (-0.08,0.09) | 0.02 |
| **Food Fussiness** | Crude | -0.01  (-0.18, 0.18) | 0.958 | **2.97**  **(2.88, 3.02)** | 0.00  (-0.09, 0.08) | 0.00 | 0.03  (-0.20, 0.27) | 0.877 | 2.95  (2.91,2.98) | 0.00  (-0.04, 0.05) | 0.00 |
|  | Adjusted^b^ | -0.03  (-0.21, -0.16) | 0.745 | **3.00**  **(2.55, 3.46)** | 0.00  (-0.08, 0.09) | 0.00 | 0.03  (-0.21, 0.27) | 0.892 | 2.97  (2.69,3.26) | -0.00  (-0.05, 0.04) | 0.00 |

CI: confidence interval. R^2^ variance explained by the model. ^a^ Food security status assessed by the US Household Food Security Survey Module (United States Department of Agriculture, 2012), completed by a parent or primary caregiver; Food secure as the reference category. ^b^ Model adjusted for maternal age, education, family structure, neighborhood deprivation, and child’s sex. Significant results in bold (*P*<0.05).

Supplementary Table 3. Agreement between children and parents reports of food security status at age 10.

|  | **Child-reported food secure ^a^** | **Child-reported food insecure** | **Total**  **n (%)** |
| --- | --- | --- | --- |
| **Caregiver-reported food secure ^b^** | 2152 | 191 | 2343 (94.2) |
| **Caregiver-reported food insecure** | 110 | 34 | 144 (5.8) |
| **Total**  **n (%)** | 2262 (90.1) | 225 (9.0) | 2487 |

^a^ Food security status assessed by the US Household Food Security Survey Module (United States Department of Agriculture, 2012), completed by a parent or primary caregiver. ^b^ Food security status assessed by the Self-Administered Food Security Survey Module (SAFSSMC) (Maia et al., 2020).

Supplementary Table 4. Associations between food security status, reported by the children at age 10, and appetitive traits at age 13.

|  |  |  | **Food insecure at age 10 ^a^** | | |  |
| --- | --- | --- | --- | --- | --- | --- |
| **Appetitive traits at age 13** |  | $\hat{\beta}$ (95% CI) | p-value | Intercept (95% CI) | Standardized $\hat{\beta}$(95% CI) | R^2^ |
| Food Responsiveness | Crude | **0.30 (0.19, 0.42)** | **<0.001** | **2.17 (2.14, 2.21)** | **0.10 (0.07, 0.14)** | 0.01 |
|  | Adjusted ^b^ | **0.28 (0.16, 0.39)** | **<0.001** | **2.76 (2.53, 3.00)** | **0.08 (0.04, 0.12)** | 0.03 |
| Enjoyment of Food | Crude | **0.20 (0.10, 0.31)** | **<0.001** | **3.23 (3.20, 3.27)** | **0.08 (0.04, 0.11)** | 0.01 |
|  | Adjusted ^b^ | **0.20 (0.09, 0.30)** | **<0.001** | **3.64 (3.42, 3.86)** | **0.07 (0.02, 0.11)** | 0.01 |
| Desire to Drink | Crude | **0.23 (0.14, 0.33)** | **<0.001** | **2.00 (1.97, 2.03)** | **0.10 (0.06, 0.14)** | 0.01 |
|  | Adjusted ^b^ | **0.18 (0.08, 0.27)** | **<0.001** | **2.60 (2.41, 2.80)** | **0.09 (0.05, 0.13)** | 0.04 |
| Emotional Overeating | Crude | **0.19 (0.09, 0.29)** | **<0.001** | **2.06 (2.03, 2.09)** | **0.07 (0.03, 0.11)** | 0.01 |
|  | Adjusted ^b^ | **0.20 (0.09, 0.30)** | **<0.001** | **2.46 (2.25, 2.67)** | **0.09 (0.05, 0.13)** | 0.03 |
| Emotional Undereating | Crude | 0.02 (-0.08, 0.13) | 0.640 | **2.28 (2.25, 2.32)** | 0.00 (-0.03, 0.05) | 0.00 |
|  | Adjusted ^b^ | 0.06 (-0.05, 0.16) | 0.293 | **2.32 (2.10, 2.53)** | 0.04 (0.00, 0.09) | 0.01 |
| Satiety Responsiveness | Crude | **-0.11 (-0.20, -0.03)** | **0.009** | **2.49 (2.46, 2.52)** | **-0.05 (-0.09, -0.01)** | 0.00 |
|  | Adjusted ^b^ | -0.08 (-0.17, 0.01) | 0.066 | **2.44 (2.26, 2.62)** | -0.02 (-0.06, 0.02) | 0.01 |
| Slowness in Eating | Crude | -0.05 (-0.17, 0.06) | 0.337 | **2.44 (2.40, 2.47)** | -0.02 (-0.06, 0.02) | 0.00 |
|  | Adjusted ^b^ | -0.03 (-0.14, 0.09) | 0.661 | **2.68 (2.45, 2.91)** | 0.02 (-0.02, 0.06) | 0.02 |
| Food Fussiness | Crude | 0.09 (-0.02, 0.20) | 0.126 | **2.94 (2.91, 2.98)** | 0.03 (-0.01, 0.07) | 0.00 |
|  | Adjusted ^b^ | 0.07 (-0.04, 0.19) | 0.201 | **2.95 (2.71, 3.18)** | 0.00 (-0.04, 0.04) | 0.00 |

CI: confidence interval. R^2^ variance explained by the model. ^a^ Food security status assessed by the Self-Administered Food Security Survey Module (SAFSSMC)(Maia et al., 2020), completed by the children; Food secure as the reference category. ^b^ Model adjusted for maternal age, education, monthly disposable income, family structure, neighbourhood deprivation, and child’s sex. Significant results in bold (*P*<0.05). N varies between 2483 to 2457.
